# Replacement of fentanyl infusion by enteral methadone decreases the weaning time from mechanical ventilation: a randomized controlled trial

**DOI:** 10.1186/cc11250

**Published:** 2012-03-15

**Authors:** Raquel Wanzuita, Luiz F Poli-de-Figueiredo, Felipe Pfuetzenreiter, Alexandre Biasi Cavalcanti, Glauco Adrieno Westphal

**Affiliations:** 1 Adult ICU, Centro Hospitalar Unimed, Rua Orestes Guimarães-905, Joinville, 89204-060, Brazil; 2 Adult ICU, Hospital Regional Hans Dieter Schmidt, Rua Xavier arp-1, Joinville, 89227-680, Brazil; 3 LIM-08, Hospital das Clínicas, University of São Paulo, Avenida Doutor Arnaldo-455, São Paulo, 01246-903, Brazil; 4 Adult ICU, Hospital Municipal São José, Avenida Getúlio Vargas-238, Joinville, 89202-000, Brazil; 5 Research Institute, Hospital do Coração, Rua Abílio Soares-250, São Paulo, 04005-000, Brazil

## Abstract

**Introduction:**

Patients undergoing mechanical ventilation (MV) are frequently administered prolonged and/or high doses of opioids which when removed can cause a withdrawal syndrome and difficulty in weaning from MV. We tested the hypothesis that the introduction of enteral methadone during weaning from sedation and analgesia in critically ill adult patients on MV would decrease the weaning time from MV.

**Methods:**

A double-blind randomized controlled trial was conducted in the adult intensive care units (ICUs) of four general hospitals in Brazil. The 75 patients, who met the criteria for weaning from MV and had been using fentanyl for more than five consecutive days, were randomized to the methadone (MG) or control group (CG). Within the first 24 hours after study enrollment, both groups received 80% of the original dose of fentanyl, the MG received enteral methadone and the CG received an enteral placebo. After the first 24 hours, the MG received an intravenous (IV) saline solution (placebo), while the CG received IV fentanyl. For both groups, the IV solution was reduced by 20% every 24 hours. The groups were compared by evaluating the MV weaning time and the duration of MV, as well as the ICU stay and the hospital stay.

**Results:**

Of the 75 patients randomized, seven were excluded and 68 were analyzed: 37 from the MG and 31 from the CG. There was a higher probability of early extubation in the MG, but the difference was not significant (hazard ratio: 1.52 (95% confidence interval (CI) 0.87 to 2.64; *P *= 0.11). The probability of successful weaning by the fifth day was significantly higher in the MG (hazard ratio: 2.64 (95% CI: 1.22 to 5.69; *P *< 0.02). Among the 54 patients who were successfully weaned (29 from the MG and 25 from the CG), the MV weaning time was significantly lower in the MG (hazard ratio: 2.06; 95% CI 1.17 to 3.63; *P *< 0.004).

**Conclusions:**

The introduction of enteral methadone during weaning from sedation and analgesia in mechanically ventilated patients resulted in a decrease in the weaning time from MV.

## Introduction

Critically ill patients are often subjected to procedures that cause pain and anxiety [1,2]. These procedures may also result in the increased activity of endogenous catecholamines, higher global and myocardial oxygen consumption, hypercoagulability and immunosuppression [3]. The use of potent analgesics, such as opioids, provides comfort and facilitates adaptation to mechanical ventilation (MV) [4,5].

Fentanyl is the preferred opioid used in many ICUs [6,7] due to its potency and its non-induction of histamine release, resulting in a low risk of inducing hemodynamic instability. However, the short duration of action of this medication often requires a continuous infusion, resulting in a rapid induction of tolerance, dependence and withdrawal syndrome possibly resulting in psychomotor agitation and/or patient-ventilator asynchrony [8-10]. The combination of midazolam and fentanyl in continuous infusion is the protocol used in most Brazilian ICUs. [7] The use of continuous infusion sedation is associated with prolongation of mechanical ventilation, longer ICU stays and longer hospital stays [11].

Several alternative treatments have emerged in the past few years that aim to reduce the duration of MV [12-15]. However, the use of opioids and benzodiazepines remains necessary in several situations, such as acute respiratory distress syndrome, intracranial hypertension or hemodynamic instability. In these situations high doses are often used for long periods, increasing the risk of acute withdrawal syndrome [8-10]. Several strategies to manage withdrawal have been utilized, including gradual weaning, replacement by another long-acting opioid, the use of opioid antagonists and the administration of adrenergic agonists, such as clonidine [16-20].

The introduction of long-acting opioids by enteral administration to prevent opioid withdrawal syndrome was first described in 1965 with the use of methadone for the rehabilitation of heroin users. Methadone reduces the drug withdrawal syndrome and thus helps to prevent relapse [21]. Since the 1970s, methadone has been used in programs regulated by the US government for the treatment of opioid dependency [22].

Several studies have demonstrated the efficacy and safety of methadone use to prevent and/or treat the withdrawal syndrome related to opioid use in pediatric ICUs [23-27]. However, there have been no similar studies conducted in adult ICUs.

The aim of this study was to test the hypothesis that the introduction of enteral methadone during weaning from sedation and analgesia in critically ill adult patients would decrease the time of weaning from MV.

## Materials and methods

A prospective, controlled, randomized, double-blind clinical trial was conducted between April 2005 and October 2009 in the adult ICUs of four general hospitals in southern Brazil.

### Participants

The subjects studied were severely ill patients who required MV and the continuous use of fentanyl and who met the following inclusion criteria: MV for at least five days, the use of fentanyl analgesia in varying doses for at least five days, or a dose of fentanyl ≥ 5 μg/kg/hour for at least 12 hours [9,26]. Subjects were also required to meet the following ventilation weaning criteria: reversal of the process that caused the respiratory failure, adequate oxygenation (PaO_2_/FiO_2 _> 200; positive end-expiratory pressure (PEEP) ≤ 5; FiO_2 _≤ 0.4 and pH ≥7.25), hemodynamic stability (with minimal or no vasoactive drugs) and neurological stability (ability to initiate respiratory effort) [28]. We excluded patients younger than 18 years, those with terminal diseases and those with cervical spinal cord injuries or neuromuscular diseases.

Patients were included in the study only after informed consent, documented as the regulations provided for in Resolution 196, October 1996, the National Board of Health. The study protocol and consent to participate in the study and consent to publish were approved by the Research Ethics Committee of Hospital Municipal São José of Joinville (Committee referenced by the other participating institutions), and recorded on 15 October 2004 under number 008/2004.

Patients were followed up from the time they met the inclusion criteria and agreed to participate in the study until death or hospital discharge.

### Interventions

The enrolled patients were randomized into two groups: the methadone group (MG), for whom intravenous fentanyl was replaced with enteral methadone; and the control group (CG), who underwent a gradual reduction of intravenous fentanyl.

The MG received the following treatment:

1. One capsule of methadone (10 mg) was administered enterally every six hours.

2. The rate of fentanyl infusion was reduced by 20% [16].

3. After 24 hours, the vial of fentanyl infusion was replaced by the study. solution (placebo) and the infusion rate was reduced by 20% [16].

4. The infusion rate for the placebo solution was reduced by 20% every 24 hours [16].

The following schedule was applied for the CG:

1. A placebo capsule was administered enterally every six hours.

2. The rate of fentanyl infusion was reduced by 20% [16].

3. After 24 hours, the vial of fentanyl was replaced by the study solution (fentanyl) and the infusion rate was reduced by 20% [16].

4. The infusion rate for the fentanyl solution was reduced by 20% every 24 hours [16].

Occasional episodes of intolerance to opioid withdrawal, characterized by agitation, anxiety, tremors, myoclonus, vomiting, diarrhea, piloerection, sweating, dilated pupils, tachycardia and hypertension, were treated with supplemental opioids as follows:

1. Bolus 1 to 2 μg/kg of fentanyl, maximum of four times in 24 hours.

2. Increase the capsule dose (methadone/placebo) by 50%.

3. Increase the infusion rate of the solution (fentanyl/placebo) to the previous dose.

The patients received benzodiazepines and antipsychotic agents by physician direction in the ICU. We did not use standard sedation scales to titrate sedation in the participant ICUs. However, the sedation goal was to keep patients calm and awake, or when not possible, slightly sedated, that is, easily arousable.

The study solutions and capsules were handled, identified and released by a pharmacist, who was the only person who knew the nature of the administered drugs. The capsules contained starch or 10 mg of methadone and were indistinguishable in appearance. The solutions contained 100 ml of saline or 50 ml of saline and 50 ml of fentanyl, also indistinguishable by sight.

We assessed readiness for weaning every morning. The ability to sustain unassisted breathing was also assessed every morning by performing a spontaneous breathing trial in patients with low levels of pressure support (≤ 10 cmH_2_O), and with other clinical and objective signs of stability. Midazolam and fentanyl were used for sedation and analgesia. Daily interruption of sedatives was not standardized in the participant ICUs. We did not have a standardized sedation scale to titrate sedation, nor did we use a uniform delirium diagnosis method. In the ICUs in the study using gradual weaning of sedation, the infusion rate was reduced by 20% every 24 hours. The purpose of this schedule was to prevent the withdrawal syndrome.

The main outcome variables assessed were the following: duration of ventilation weaning (weaning success was defined as 48 hours without reinstitution of MV) [29], duration of MV, length of ICU stay and length of hospital stay. The clinical and demographic data collected included age, sex, weight, indications for hospitalization, comorbidities (hypertension, diabetes mellitus, chronic lung disease, psychiatric and neurological disorders, obesity, alcoholism, smoking or illicit drug use), the information necessary to calculate the APACHE II (Acute Physiology And Chronic Health Evaluation) score and deaths before and after discharge from the ICU [30]. We also recorded the following factors: need for supplemental doses of fentanyl for the treatment of opioid withdrawal intolerance, accumulated doses of fentanyl and midazolam maleate used before the start of weaning and the use of other sedatives.

### Randomization

The randomization between the two groups (MG and CG) was performed by a simple lottery system; 75 pieces of paper with the word yes and the same number with the word no were placed in a package. The sequence was determined by drawing, and was recorded on a specific form to be used by the pharmacist. Only the pharmacist from the hospital at which the randomization process occurred knew the nature of the administered medications.

### Sample size and statistical methods

The sample size was determined as 70 patients, calculated from data obtained in a pilot study (23 patients) that preceded this study. For this calculation, the time-to-event analysis was used, with a cumulative probability of successful extubation on the fifth day in 20% of the CG and 50% of the MG. We applied a significance level of 5% and power of 80%.

For the statistical processing of the data, we used NCSS Statistical Software 2007, PASS 11 (Power Analysis and Sample Size) and GraphPad Prism 4. Continuous variables are shown as the means ± standard deviation and were compared using a Student's t-test. Categorical variables are expressed as their absolute and relative values and were compared using a chi-squared test. A *P*-value < 0.05 was considered statistically significant. To test the effects of two therapeutic regimens on the duration of weaning, the duration of MV and the length ICU stay, we used time-to-event analysis. These data were presented as Kaplan-Meier curves, and for comparison between groups, a Cox regression (Cox proportional hazards model) was used. Patients who died were censored at the time of death. The time of weaning was also assessed, taking into consideration only the patients who were permanently disconnected from MV. For questions inherent to the study methodology, the interruption of the fentanyl infusion in patients from the CG occurred only on the fifth day; for this reason, we also evaluated the weaning period in the interval between randomization and the fifth day. For the analysis of MV-free days within 28 days after the start of the protocol, the median, interquartile range (IQR) and Wilcoxon rank-sum (Mann-Whitney test) were used when comparing the groups.

## Results

**Figure 1:**
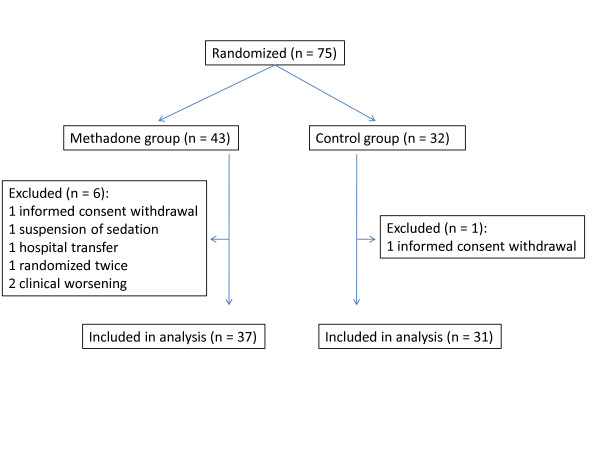
**Study flowchart**.

Between the months of April 2005 and October 2009, 75 patients were randomized at the four ICUs: 43 to the MG and 32 to the CG. Of these, seven patients were excluded (two informed consent withdrawal, one suspension of sedation, one hospital transfer, one randomized twice, two clinical worsening) and 68 patients were included in the analysis: 37 in the MG and 31 in the CG (Figure 1). The patients were monitored until discharge or death. All patients were analyzed by the intention-to-treat method.

Patients in both groups had similar demographic and clinical characteristics (Table 1).

**Table 1:** Demographic and clinical characteristics.

Characteristics	MG (*n *= 37)	CG (*n *= 31)	*P* value	
Males, number (%)	27 (72)	26 (83)	0.28	
Age	43 ± 18	45 ± 17	0.60	
Weight	82 ± 16	84 ± 19	0.58	
APACHE II	19 ± 8	18 ± 6	0.59	
Cause of hospitalization, n (%)				
Trauma	17 (45)	14 (45)	0.94	
Respiratory	6 (16)	3 (9)	0.42	
Sepsis	5 (13)	7 (22)	0.32	
Abdominal surgery	5 (13)	2 (6)	0.33	
Neurological	3 (8)	3 (9)	0.82	
Exogenous intoxication	0	2 (6)	0.11	
Cardiac	1 (2)	0	0.35	
Fentanyl Initial dose (μg/kg/h)	2.15 ± 1.06	2.35 ± 1.21	0.48	
Accumulated dose (μg)	40,608 ± 24,882	41,284 ± 20,545	0.90	
Midazolam maleate				
Accumulated dose (mg)	1,052 ± 820	1,014 ± 672	0.83	
Days on fentanyl before start of weaning	10.1 ± 6	10.4 ± 4.4	0.80	
MV days before start of weaning	10.6 ± 6.8	10.3 ± 4.5	0.87	
ΔT Hosp. X ICU	3 ± 8	4 ± 12	0.88	

Data are presented as n (%) or mean ± standard deviation. ΔT Hosp. X ICU = Time from hospital stay until ICU admission. APACHE, acute physiology and chronic health evaluation; CG, Control group; ICU, intensive care unit; MG, methadone group; MV, mechanical ventilation

**Figure 2:**
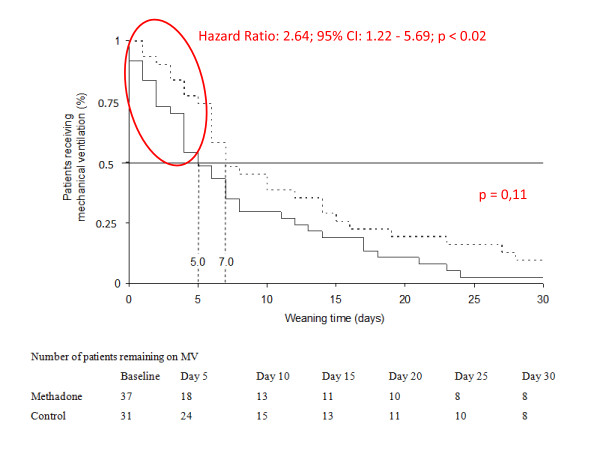
**Kaplan-Meier curve of the weaning time from MV**. The curve shows the probability of successful weaning (*P *= 0.11). The difference was significant until the fifth day (*P *< 0.02). Dotted line: CG (parenteral fentanyl); continuous line: MG. The numbers on the perpendicular dotted lines are the median durations of the weaning time in each group. CG, control group; MG, methadone group; MV, mechanical ventilation.

**Figure 3:**
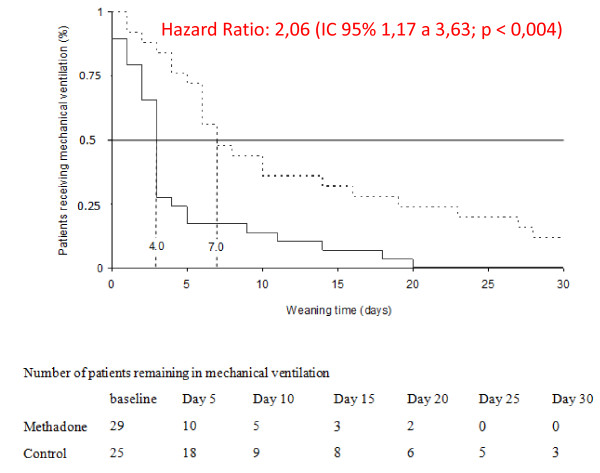
**Kaplan-Meier curve of the weaning time from MV among the 54 patients who were successfully weaned**. The curve shows the probability of successful weaning from MV (*P *< 0.004). Dotted line: CG (parenteral fentanyl); continuous line: MG. The numbers on the perpendicular dotted lines are the median durations of the weaning time in each group. CG, control group; MG, methadone group; MV, mechanical ventilation.

There were no significant differences between the groups when we analyzed the weaning time of the 68 patients (Hazard ratio: 1.52; 95% CI: 0.87 to 2.64; *P *= 0.11). Analyzing the interval between randomization and the fifth day of weaning, the probability of successful weaning was significantly higher in the MG (Hazard ratio: 2.64; 95% CI: 1.22 to 5.69; *P *< 0.02). The median weaning time was five days in the MG (95% CI: 2.42 to 7.58), and in the CG, this interval was seven days (95% CI: 2.87 to 11.13) (Figure 2). Of the 68 patients included in the analysis (MG: 37 versus CG: 31), 14 died before completing the ventilation weaning (Table 2). Among the 54 survivors (MG: 29 versus CG: 25), the time of MV weaning was significantly lower in the MG (Hazard ratio: 2.06; 95% CI: 1.17 to 3.63; *P *< 0.004). The median weaning time was four days for the MG (95% CI: 1.99 to 6.01) and seven days (95% CI: 2.5 to 11.5) for the CG (Figure 3).

**Table 2:** Patients who completed weaning.

Patients	Total	Methadone Group	Control Group
Included	68	37	31
Completed weaning	54	29	25
Censored at death	14	08	06
Total death during the study	24	12	12

Analyzing the MV-free days after 28 days from the beginning of the protocol, we observed a trend favoring the MG, with a median of 20 days (7 to 24) versus16 days (0 to 22) in the CG (*P *= 0.13).

There were no significant differences in the total duration of MV between the groups (hazard ratio 1.54; 95% CI: 0.85 to 2.81; *P *= 0.14). The median time of MV in the MG was 15 days (95% CI: 11.03 to 18.97) and in the CG, this interval was 20 days (95% CI: 15.25 to 24.75). The duration of the ICU stay did not differ between the groups (hazard ratio 1.47; 95% CI: 0.81 to 2.66, *P *= 0.19) and was 19 days (95% CI: 14.23 to 23.77) in the MG and 25 days (95% CI: 20.37 to 29.63) in the CG.

The groups also did not differ regarding the length of hospital stay (hazard ratio 1.14; 95% CI: 0.65 to 2.00, *P *= 0.62), with a median of 44 days (95% CI: 33.67 to 54.33) in the MG and 47 days (95% CI: 33.67 to 54.33) in the CG.

The cumulative dose of fentanyl received before the start of weaning was similar in both groups (MG: 40,608 ± 24,882 versus 41,284 ± 20,545 μg, *P *= 0.90). In the MG, 10 patients showed signs of opioid withdrawal intolerance compared to 12 patients in the CG (*P *= 0.30). Patients in both groups used other drugs for sedation, analgesia and delirium treatment during the ICU stay; 29 were from the MG and 27 were from the CG (*P *= 0.34).

The groups were also similar with regard to the presence of comorbidities (*P *= 0.75), the development of complications during the ICU stay (*P *= 0.85) and the number of patients undergoing tracheostomy (*P *= 0.67).

The hospital mortality rate was similar between the groups (MG: 12/37 versus CG: 12/31, *P *= 0.58). Of the 24 deaths, 9 occurred after discharge from the ICU. Among the 15 patients who died during the ICU stay, 14 did not complete MV weaning (MG: 8/27 versus CG: 6/31, *P *= 0.81).

## Discussion

This study compared the effects of a gradual reduction of intravenous fentanyl versus the replacement of fentanyl by enteral methadone during the weaning from sedation and analgesia in patients undergoing MV. The results demonstrated that the substitution of intravenous fentanyl with enteral methadone shortens the time of ventilation weaning in critically ill adult patients undergoing prolonged sedation and analgesia.

The overall analysis of the weaning time from MV showed no differences between the groups analyzed by this study. However, the chance of successful weaning by the fifth day was significantly higher in the MG (Figure 2). This time interval was adopted based on a pilot study that preceded this study and informed the sample size calculation. In this calculation, the fifth day after inclusion in the protocol was identified as the threshold for ensuring the success of weaning. Rozendaal *et al. *used a combination of remifentanyl and propofol to reduce the time of ventilation weaning and reported that the effect of this combination on weaning time was time-dependent, as we observed in the methadone group [31].

During this five-day period, the CG continued to receive fentanyl, a situation that may have contributed to the prolongation of ventilation weaning. The benefit of this strategy of fentanyl substitution during the weaning period is probably due to the decrease in time required to remove the intravenous infusion of fentanyl. In the MG, the complete withdrawal of fentanyl occurred after 24 hours with all patients. However, in the CG, the gradual withdrawal of fentanyl resulted in the persistence of this drug infusion for at least five days. Thus, it is possible that the action of this potent opioid may have hindered the final disconnection from MV in the CG. It is possible that a more aggressive reduction of fentanyl in the control group, for example daily fentanyl interruption returning the drug only in case of signs of opioid withdrawal, might have accelerated weaning in the control group.

By focusing the analysis on the 54 patients who completed the MV weaning, we observed that the group receiving enteral methadone as a substitute for fentanyl benefitted by the reduction in the time of weaning from MV (Figure 3). However, interpretation of this data should be very cautious because it is vulnerable to attrition bias: non-weaned patients were excluded, and we cannot completely reject an association between the intervention (methadone) and the reason for exclusion (death or long-term mechanical ventilation need).

Opioid substitution strategies aim to suppress the opioid withdrawal intolerance that, as demonstrated in children in the study of Katz *et al.*, is correlated to the total amount received and the infusion duration. Accumulated doses of 1.5 mg/kg fentanyl or a continuous infusion over five days is associated with more than a 50% chance of developing a withdrawal syndrome. This rate increases to 100% at doses of 2.5 mg/kg or an infusion duration of nine days or more [9]. The abrupt withdrawal of fentanyl may be associated with manifestations of withdrawal that necessitate the administration of supplemental doses of parenteral opioids, in addition to sedatives and antipsychotics. Such procedures may result in respiratory depression or impairment of the cough reflex, which compromise the safety of extubation and result in longer MV.

The concern with early ventilation weaning is justified by the complications associated with extensions in MV duration, ICU stay and hospital stay [32]. In this context, the substitution of fentanyl with methadone may be a viable management strategy for ventilation weaning.

Case series and historical control studies in pediatric intensive care units have suggested a role for methadone in preventing opioid withdrawal syndrome and accelerating IV opioid weaning [23-27]. We have not found any study using methadone in adult ICUs.

The replacement of opioid agonists with clonidine is also effective in the treatment and prevention of withdrawal symptoms; however, side effects, such as bradycardia and hypotension, may limit its use [18,20]. Other strategies, including the use of extremely short duration opioids (remifentanil) and propofol, have resulted in a decreased duration of MV and ICU stay [31,33].

The use of enteral methadone did not reduce the duration of MV or the ICU or hospital stay, as has been observed in other strategies for the abbreviation of the duration of MV using alternative protocols for sedation and analgesia [12-15]. Several factors may have contributed to this result. First, our sample was calculated to evaluate the length of MV weaning. Possibly, a study with a larger sample size and a different design might better assess the impact of this strategy on the duration of MV, ICU stay and hospital stay.

Our study had some other limitations. The randomization was performed by a simple lottery system. Perhaps we should have used a block randomization scheme. The prolonged phase of patient enrollment may have generated bias. The difficulty in recognizing and distinguishing symptoms of withdrawal from expressions of pain is also potentially significant in this study. All adrenergic manifestations were treated with a bolus of fentanyl, and at the discretion of the ICU doctor, a bolus of midazolam was also added. Unfortunately, we have not collected data on the dose of other sedatives, analgesics and antipsychotics.

## Conclusions

The results of this study suggest that the introduction of enteral methadone during the weaning process from sedation and analgesia for mechanically ventilated patients may abbreviate the duration of weaning from MV. We believe these results need confirmation by larger randomized trials before being considered for clinical practice.

## Key messages

• Abrupt interruption of IV opioids after more than five days of continuous infusion may lead to withdrawal manifestations; however, gradual tapering of fentanyl may delay extubation.

• Replacement of fentanyl by enteral methadone in critically ill adult patients who were on fentanyl infusion for more than five days may abbreviate weaning from mechanical ventilation without increasing withdrawal manifestations.

• Larger randomized controlled trials are required to confirm these observations.

## Abbreviations

APACHE: acute physiology and chronic health evaluation; CG: control group; CI: confidence interval; ICU: intensive care unit; IV: intravenous; MG: methadone group; MV: mechanical ventilation; PEEP: positive end-expiratory pressure.

## Competing interests

The authors declare that they have no competing interests.

## Authors' contributions

RW conceived of the study, participated in its design and coordination, and helped to draft the manuscript. LFPF participated in the design and coordination of the study. GAW conceived of the study, participated in its design and coordination, and helped to draft the manuscript. FP participated in the design of the study and helped to draft the manuscript. ABC participated in the design of the study and performed the statistical analysis. All authors read and approved the final manuscript.

## Authors' information

Raquel Wanzuita is a PhD student at the Graduate Program in Anesthesiology of Faculty of Medicine of the University of São Paulo (FMUSP) - Strict Sense.

Luiz Francisco Poli de Figueiredo is a teacher advisor at the Graduate Program in Anesthesiology of Faculty of Medicine of the University of São Paulo (FMUSP) - Strict Sense.
